# Computational analysis and verification of molecular genetic targets for glioblastoma

**DOI:** 10.1042/BSR20201401

**Published:** 2020-06-16

**Authors:** Liang Xue, Haibing Liu, Yehuang Chen, Liangfeng Wei, Jingfang Hong

**Affiliations:** Department of Neurosurgery, 900 Hospital of the Joint Logistics Team, Fuzhou, Fujian 350025, P.R. China

**Keywords:** Differentially expressed genes, Glioblastoma, Hub gene, ifferentially expressed miRNAs, Protein-protein interaction

## Abstract

Background: Glioblastoma (GBM) is the most common malignant brain tumor with a poor prognosis. The initial treatment for high-grade gliomas is surgical excision. However, even with concomitant use of radiation or chemotherapy, patients are still prone to recurrence. The specific pathogenesis of GBM is still controversial.

Methods: Differentially expressed genes (DEGs) and differentially expressed miRNAs (DEMs) between GBM and normal brain tissues were screened. *P*-value was obtained by Bayes test based on the limma package. Statistical significance was set as *P*-value <0.05 and |Fold change (FC)| > 0.2 (GSE90886); *P*-value <0.05 and |FC| > 1 (GSE116520, GSE103228). Gene Ontology (GO) analysis, Kyoto Encyclopedia of Genes and Genomes (KEGG), protein–protein interaction (PPI) network were performed. Hub genes were selected from miRNA target genes and DEGs. GBM and normal brain tissues were extracted to verify the expression.

Results: A total of 100 DEGs were overlapped in both datasets. Analysis of pathways and process enrichment tests indicated that ion transport, positive regulation of macromolecule metabolic process, cell cycle, axon guidance were enriched in the GBM. Sixteen hub genes were identified. Hub genes ADARB1 and neuropilin 1 (NRP1) were significantly associated with overall survival (OS) and disease-free survival (DFS) (*P*<0.05). Eukaryotic translation termination factor 1 (ETF1) was associated with DFS (*P*<0.05).

Conclusions: DEGs and DEMs were found between GBM tumor tissues and normal brain tissues. These biomarkers may be used as targets for early diagnosis and specific treatment.

## Introduction

Glioblastoma (GBM) is a common intracranial tumor of the central nervous system (CNS) in adults, with high malignancy and rapid rate of progression [1]. A recent report based on the Central Brain Tumor Registry of the United States (CBTRUS) showed that GBM accounts for approximately 14.6% of all brain tumors [2]. In addition, the average annual age-adjusted incidence rate (AAAIR) of the CNS tumors is 7.08 per 100000 population [2]. The symptoms of patients mainly depend on the location and size of the tumor [3]. Patients may experience headaches and vomiting due to increased intracranial pressure. Invasion of tumor in the cranial cavity can cause symptoms such as hemiplegia, hemianopia, and aphasia [4]. GBMs often progress rapidly with poor prognosis. After the diagnosis of GBM, the 5-year relative survival rate is 35.8% [2]. In fact, early diagnosis and treatment are necessary. Surgical resection is the most important treatment for high-grade gliomas. Maximum resection of the tumor while preserving neurological function is crucial [5]. However, even with concomitant use of radiation or chemotherapy, patients are still prone to recurrence [4]. At present, the specific pathogenesis of GBM still remains controversial. The promoter methylation of methyl guanine methyl transferase (MGMT) [6], mutations in isocitrate dehydrogenase 1 (IDH1) or dehydrogenase 2 (IDH2), and other mechanisms are all involved in the generation and development of GBM [7,8]. Moreover, there is evidence showing that small molecules and pathways were involved in the development of GBM. For example, RNA regulatory factors can regulate the activity of microglia and participate in tumor progression and can act as a therapeutic target in GBM [9]. Runt-related transcription factor 1 (RUNX1) would impact the prognosis of GBM via the TGFβ pathway [10]. Therefore, it is important to further study the molecular mechanism of GBM and find targets for more effective early diagnosis and specific therapy.

Bioinformatics analysis technology is widely used to find genetic changes in the process of tumorigenesis and development. It is a reliable method for finding diagnostic and therapeutic targets. Zhang et al. analyzed the genome-wide miRNA profile microarray data of patients with gastric cancer and found relevant molecules miR-19b-3p and miR-16-5p, which provided new ideas for the diagnosis and treatment of gastric cancer [11]. Zhang et al. found that CD276 may be involved in the progression of GBM by affecting protein phosphorylation and regulating the TGF-β pathway through bioinformatics analysis [12]. CD276 may be a suitable therapeutic target for GBM [12]. Exploring accurate molecular targets for the occurrence and progression of GBM is of great value. However, there could be false positives in data analysis. Comprehensive analysis and repeated verification of large-size samples with multiple datasets can improve the accuracy of the results.

Differentially expressed genes (DEGs) and differentially expressed miRNAs (DEMs) between GBM tumor tissues and normal brain tissues were screened by bioinformatics analysis. Gene Ontology (GO) analysis and Kyoto Encyclopedia of Genes and Genomes (KEGG) analysis were performed on DEGs. A protein–protein interaction (PPI) network was also constructed for DEGs, and miRNA-related target genes were predicted. Hub genes were selected from miRNA target genes and DEGs, and the expression of hub genes was analyzed with GBM data in the The Cancer Genome Atlas (TCGA) database. Survival analysis was performed to find the candidate hub genes. Furthermore, we used GBM tissues and normal brain tissues to confirm the expression of the hub gene and related miRNAs. Finally, we made a preliminary analysis of the DEGs role in GBM.

## Materials and methods

### GEO dataset

The GEO (http://www.ncbi.nlm.nih.gov/geo) is a public platform for the storage of gene data [13]. Two expression profiling datasets [GSE90886 (GPL15207 platform), GSE116520 (GPL10558 platform)] and one microRNA expression profiling dataset [GSE103228 (GPL18058 platform)] were, respectively, downloaded from the GEO database. The GSE90886 dataset includes nine GBM samples and nine normal control samples collected from epilepsy surgery.

The GSE116520 dataset consists of 17 GBM tissue samples, 8 normal brain tissue samples, and 17 peritumoral brain zone tissues. We only chose 17 GBM tissue samples, 8 normal brain tissue samples from the GSE116520 dataset based on the source type. The GSE103228 dataset contains 5 GBM samples and 5 normal control samples.

### DEGs identified using GEO2R

GEO2R (https://www.ncbi.nlm.nih.gov/geo/geo2r/) is an effective online tool used to identify DEGs in datasets from the GEO [14]. GEO2R may also be used to distinguish DEGs and DEMs in GBM and normal tissue samples. *P*-value was obtained by Bayes test based on the limma package. Statistical significance: *P*-value<0.05 and |Fold change (FC)| > 0.2 (GSE90886); *P*-value <0.05 and |FC| > 1 (GSE116520, GSE103228). Volcano diagrams were delineated by SangerBox software (http://sangerbox.com/). Venn diagram was obtained by FunRich software (http://www.funrich.org).

### Pathway enrichment analysis of DEGs

The Database for Annotation, Visualization and Integrated Discovery (DAVID) (https://david.ncifcrf.gov/home.jsp; version 6.8) is an online suite of analysis tools with an integrated discovery and annotation function [15]. The GO resource includes biological process (BP), cellular component (CC), and molecular function (MF). To perform the GO and KEGG analysis of DEGs, the online tool of DAVID was implemented. *P*-value was obtained by Fisher Exact Statistics. Statistical significance was defined as *P*<0.05. Metascape was used to perform the pathway and process enrichment. Gene Prioritization by Evidence Counting (GPEC) is an algorithm we are developing to identify a subset of input genes that are more likely to be the true hits. The best scoring *P*-values from the original gene lists and derived gene lists were chosen as the GPEC *P*-value of the term [16].

### PPI network

Search Tool for the Retrieval of Interacting Genes (STRING) (http://string.embl.de/) was applied to construct a PPI network of the identified DEGs. Cytoscape visualization software (version 3.6.1) was used to present the network [17]. We chose a confidence score >0.4 as the judgment criterion.

### Identification and analysis of hub genes

miRNet (http://www.mirnet.ca) includes data on the interaction of miRNAs with target genes [18]. We took the intersection of the GBM-related miRNA and DEMs which were predicted by miRNet. While, the five miRNAs which had the most significant up-regulation and down-regulation on DEMs were employed. The target genes for these miRNAs were predicted by miRNet. Then, the target genes for the miRNAs were intersected with DEGs. The obtained intersection genes were considered as hub genes. In addition, the GO analysis of hub genes was performed with Metascape. PPI was made with STRING. Coexpedia (www.coexpedia.org), a powerful co-expression analysis tool, was applied for gene co-expression analysis [19].

### Expression analysis of hub genes and survival analysis

UCSC Xena (https://xena.ucsc.edu/welcome-to-ucsc-xena/) was engaged in integrating the public genomic datasets to analyze the expression level of hub genes. Then, the clustering analysis of the expression level of hub genes was performed with heatmaps. Then GEPIA, a web server for cancer and normal gene expression profiling and interactive analyses (http://gepia.cancer-pku.cn/) [20] was applied for survival analysis of hub genes. The hazard ratio was calculated based on the Cox PH Model. Statistical analysis method applied was the Log-rank test, also known as Mantel–Cox test. Meantime, GEPIA was employed for confirming the expression of hub genes and the median expression of tumor and normal samples in bodymap again. The method for DEGs analysis was the one-way ANOVA. The expression on Box Plots |Log_2_FC| cutoff is 1 and *P*-value cutoff is 0.01, all of which matched the TCGA data. cBioPortal is useful in integrative analysis of cancer genes and clinical information (http://cbioportal.org) [21]. We used cBioportal to investigate the hub gene in Genomic Alteration Types and putative copy-number alterations from GISTIC. Brain RNA-seq is an RNA-sequencing transcriptome and splicing database of glia, neurons, and vascular cells of the cerebral cortex (http://web.stanford.edu/group/barres_lab/brain_rnaseq.html) [22]. We performed the expression and location of hub genes in cerebral cells with this tool.

### Verification of hub genes and miRNAs

A total of five participants with GBM WHO grade IV and five patients having cortex surgery due to epilepsy were recruited. After the surgeries, five GBM tumor samples from GBM patients and five normal brain tissue samples were obtained. The research conformed to the Declaration of Helsinki and was authorized by the Human Ethics and Research Ethics Committees of the 900 Hospital of the Joint Logistics Team. Informed consents had been obtained from all participants. Briefly, RNA was extracted from five tumor samples and five normal tissue samples with the RNAiso Plus (TRIzol) kit (Thermo Fisher), and was reverse transcribed to cDNA. Real-time quantitative polymerase chain reaction (RT-qPCR) was performed using specific primers for genes. The primer sequences used in the experiments were shown in Table 1. GAPDH was used as an endogenous control. *P*-value was obtained by one-way ANOVA. In addition, we verified the expression of eukaryotic translation termination factor 1 (ETF1) and neuropilin 1 (NRP1) proteins in GBM and normal brain tissues by Western blot. Furthermore, miRNA reverse transcription was performed with the miScriptII Reverse Transcription kit (Qiagen, cat. 218161) according to instructions. The cDNAs obtained in this procedure were further amplified by quantitative PCR (qPCR) with the miScript SYBR Green PCR kit. Forward miRNA specific primers used are shown in Table 1. The reverse primer of Universal Primer (UP) was used in all amplifications. The miRNAs amplification was performed with U6 RNA amplification levels. MiRNet predicted that NRP1 was a putative target for hsa-mir-218-5p and ETF1 was a target for hsa-mir-128-3p. Luciferase activity analysis was also carried out by using Dual-Luciferase Reporter Assay System (Promega).

**Table 1:** Primers and their sequences for analysis

Primer	Sequence (5′–3′)
ETF1 forward	CACGAGTGGCAAAAATGTTAGC
ETF1 reverse	CCAGGACTGAAAGGCGGTTTA
NPR1 forward	CTTCGGTGTCAAGGACGAGTA
NPR1 reverse	GGTAGGCGTAGAGCATGAGC
GAPDH forward	TGTGGGCATCAATGGATTTGG
GAPDH reverse	ACACCATGTATTCCGGGTCAAT
miR-128-3p forward	TCACAGTGAACCGGTCTCTTT
miR-128-3p reverse	CAGGTCCAGTTTTTTTTTTTTTT
miR-218-5p forward	AACACGAACTAGATTGGTACA
miR-218-5p reverse	AGTCTCAGGGTCCGAGGTATT
U6 forward	CTCGCTTCGGCAGCACA
U6 reverse	AACGCTTCACGAATTTGCGT

### Predicted lncRNAs and transcription factors

We predicted the miRNA-related lncRNA with miRNet and the miRNAs-related transcription factors (TFs) with TransmiR v2.0, an updated TF–microRNA interaction database (http://www.cuilab.cn/transmir) [23]. A diagram showing the functions of lncRNA–miRNA–mRNA was provided with Cytoscape.

## Results

### Screening of DEGs and DEMs in GBM and normal brain tissues

**Figure 1:**
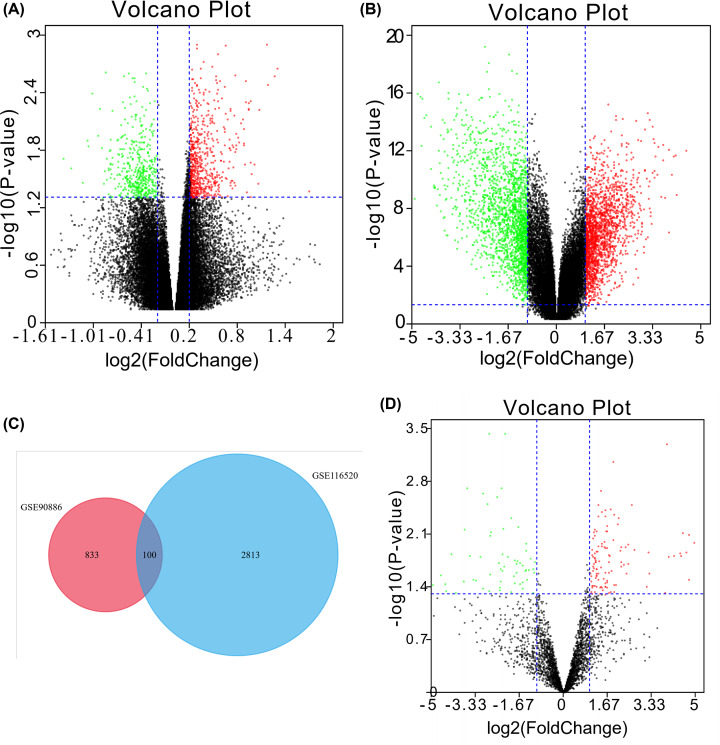
Identification of DEGs and DEMs between GBM and normal brain samples (**A**) The DEGs between GBM and normal brain samples in the GSE90886 were presented in the volcano plots, in which the green nodes mean the down-regulated DEGs, and the red nodes mean the up-regulated DEGs. (**B**) The DEGs between GBM and normal brain samples in the GSE116520 were presented in the volcano plots. (**C**) The Venn diagram manifested 100 DEGs that were common to both datasets. (**D**) The DEMs between GBM and normal brain samples in the GSE103228 were presented in the volcano plots, in which the green nodes mean the down-regulated DEMs, and the red nodes mean the up-regulated DEMs.

Volcano diagrams showed the DEGs and DEMs (Figure 1A,B,D). Twenty DEMs with the most significant up-regulation and the down-regulation were shown in Table 2. A Venn diagram revealed 100 common DEGs in the two datasets (Figure 1C).

**Table 2:** The top ten up-regulated and down-regulated miRNAs

MiRNA	Change	LogFC	*P*-value
Has-mir-148a-3p	Up-regulated	6.29	0.012
Has-mir-590-5p	Up-regulated	5.74	0.022
Has-mir-455-3p	Up-regulated	5.47	0.01
Has-mir-310b	Up-regulated	4.95	0.01
Has-mir-20a-3p	Up-regulated	4.75	0.008
Has-mir-494	Up-regulated	4.45	0.015
Has-mir-4692	Up-regulated	3.97	0.015
Has-mir-21-3p	Up-regulated	3.9	0.0005
Has-mir-3529-3p	Up-regulated	3.26	0.026
Has-mir-5681a	Up-regulated	3.21	0.013
hsa-miR-873-5p	Down-regulated	−5.26	1.91E-05
hsa-miR-218-5p	Down-regulated	−4.64	0.027
hsa-miR-144-5p	Down-regulated	−4.5	0.036
hsa-miR-136-5p	Down-regulated	−4.45	0.044
hsa-miR-29c-5p	Down-regulated	−4.25	0.014
Has-mir-129-5p	Down-regulated	−3.76	0.0003
Has-mir-3200-3p	Down-regulated	−3.74	0.007
Has-mir-128	Down-regulated	−3.64	0.002
Has-mir-544a	Down-regulated	−3.56	0.152
Has-mir-19b-2-5p	Down-regulated	−3.41	0.0318

### Functional annotation for DEGs using KEGG and GO analysis

**Figure 2:**
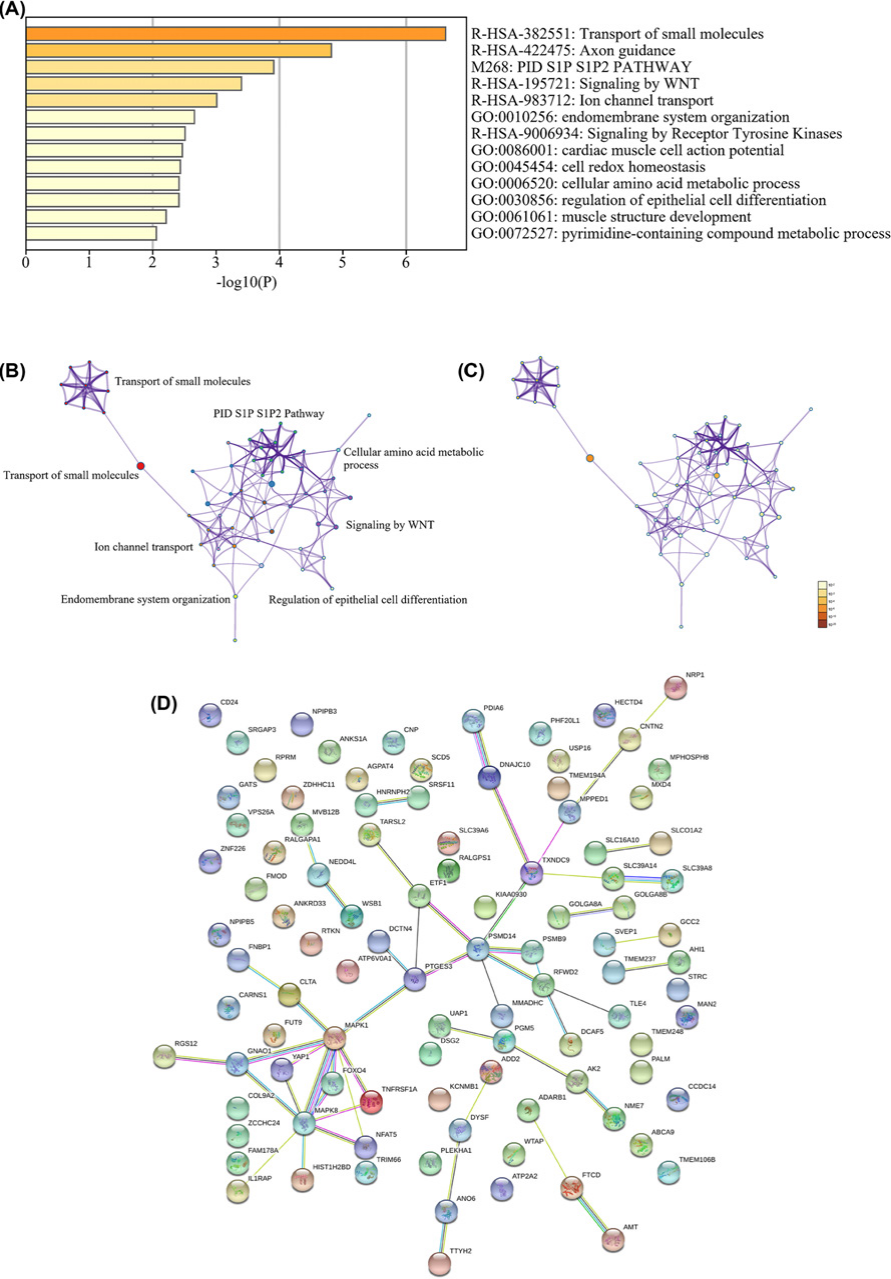
The enrichment analysis of DEGs by Metascape and PPI network (**A**) Bar graph of enriched terms across DEGs, colored by *P*-values. (**B**) Network of enriched terms, colored by cluster. (**C**) Network of enriched terms, colored by significant *P*-value. (**D**) PPI network.

The results of GO analysis revealed that variations in the BP were predominantly enriched in ion transport, positive regulation of macromolecule metabolic process, cell cycle, and so on. Variations in MF were commonly seen in neuron development, and negative regulation of cell differentiation (Table 3). KEGG analysis demonstrated that DEGs were widely discovered in axon guidance, one carbon pool by folate (Table 3). Pathways and process enrichment analyses by Metascape were shown in Figure 2A–C.

**Table 3:** GO and KEGG pathway enrichment analysis of DEGs in GBM samples

Term	Description	Count in gene set	*P*-value
GO:0006811	Ion transport	11	0.0043
GO:0010604	Positive regulation of macromolecule metabolic process	9	0.0602
GO:0007049	Cell cycle	8	0.0883
GO:0031175	Neuron projection development	5	0.0379
GO:0048666	Neuron development	5	0.0871
GO:0045596	Negative regulation of cell differentiation	4	0.0916
GO:0042470	Melanosome	3	0.0732
GO:0048770	Pigment granule	3	0.0732
GO:0009898	Internal side of plasma membrane	5	0.0734
GO:0004707	MAP kinase activity	2	0.07
GO:0046873	Metal ion transmembrane transporter activity	5	0.0891
hsa04360	Axon guidance	4	0.0463
hsa00670	One carbon pool by folate	2	0.0962

### Construction of the PPI network

Construction of a PPI network revealed 54 edges and 51 nodes in the PPI network (PPI enrichment; Figure 2D). The network possessed significantly more interactions than expected. Such enrichment indicated that the identified proteins were at least partially associated with the pathway.

### Selection and functional annotation for Hub genes

**Figure 3:**
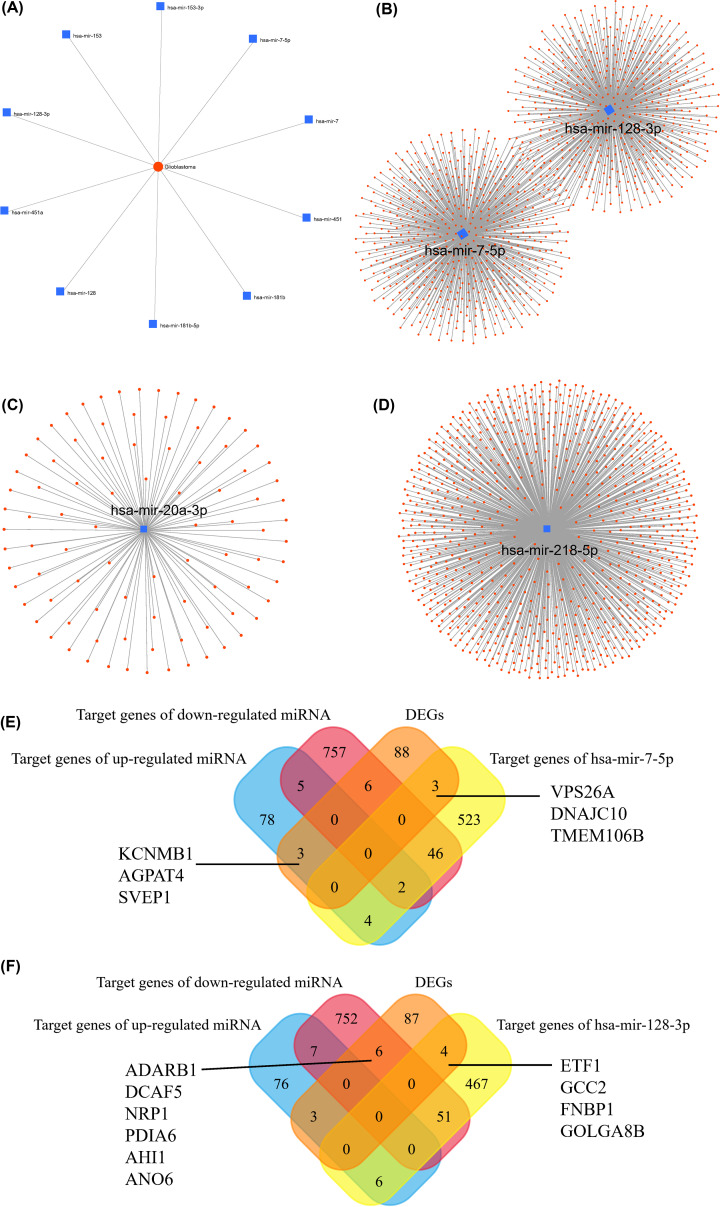
MiRNA target genes and hub genes selection (**A**) MiRNet predicted GBM-related genes. (**B**) hsa-mir-7-5p and hsa-mir-128-3p targeted gene. (**C**) hsa-mir-20a-3p targeted gene. (**D**) hsa-mir-218-5p targeted gene. (**E**) The Venn diagram manifested six genes that were common to DEGs and miRNA target genes. (**F**) The Venn diagram manifested ten genes that were common to DEGs and miRNA target genes.

**Figure 4:**
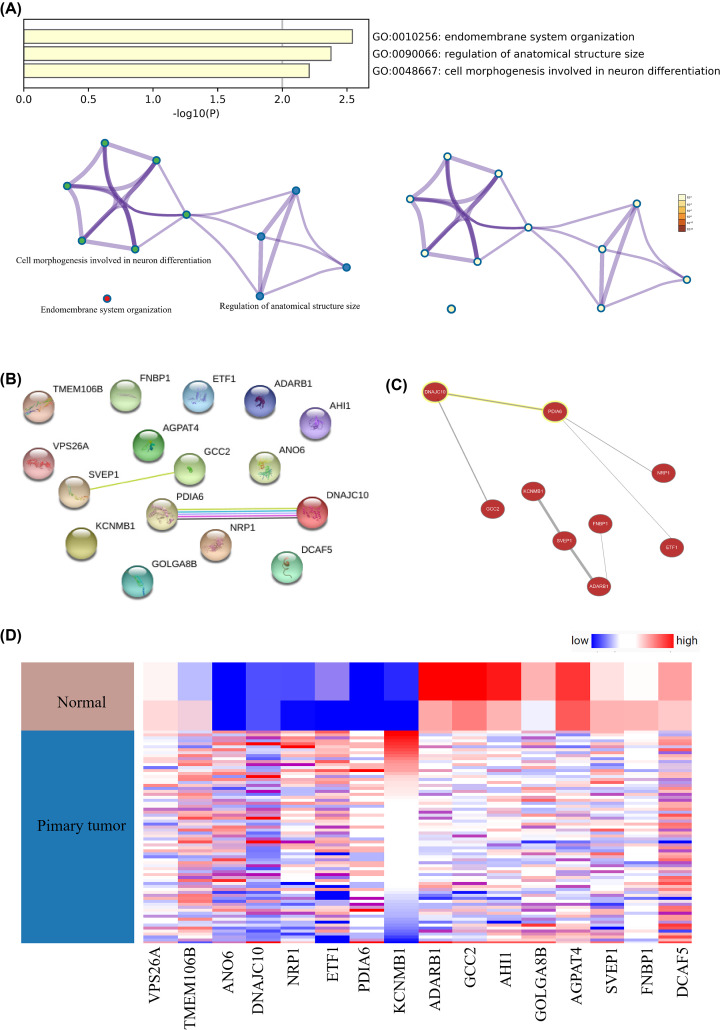
The enrichment analysis and co-expression of hub genes (**A**) Enrichment analysis of hub genes. (**B**) PPI network of hub genes. (**C**) Co-expression of hub genes. (**D**) UCSC analysis the expression of hub genes.

After using miRNet to predict the ten miRNAs most related to GBM as shown in Figure 3A, the two down-regulated miRNAs, hsa-mir-7-5p and hsa-mir-128-3p, were found as the intersection with DEMs. The genes related to them were predicted as indicated in Figure 3B. The target genes for up-regulated miRNA (hsa-mir-20a-3p) and down-regulated miRNA (hsa-mir-218-5p) were found (Figure 3C,D). The Venn diagram was applied to obtain the intersection of the miRNA target gene and DEGs. The intersection genes were the hub gene. Sixteen hub genes, KCNMB1, AGPAT4, SVEP1, ADARB1, DCAF5, NRP1, PDIA6, AHI1, ANO6, VPS26A, DNAJC10, TMEM106B, ETF1, GCC2, FNBP1, GOLGA8B, were presented in Figure 3E,F. Then the gene enrichment analysis of the hub genes found that these genes were mainly concentrated in the membrane system and regulation of cell morphogenesis involved in neuron differentiation (Figure 4A). The analysis of PPI interaction and co-expression network were provided in Figure 4B,C.

### Analysis of hub genes

The expression level of hub genes and the clustering analysis of expression level of hub genes indicated that some hub genes were higher in GBM tumor tissues, yet some other hub genes were higher in normal brain tissues (Figure 4D).

### Overall survival rate analysis and disease-free survival rate analysis

**Figure 5:**
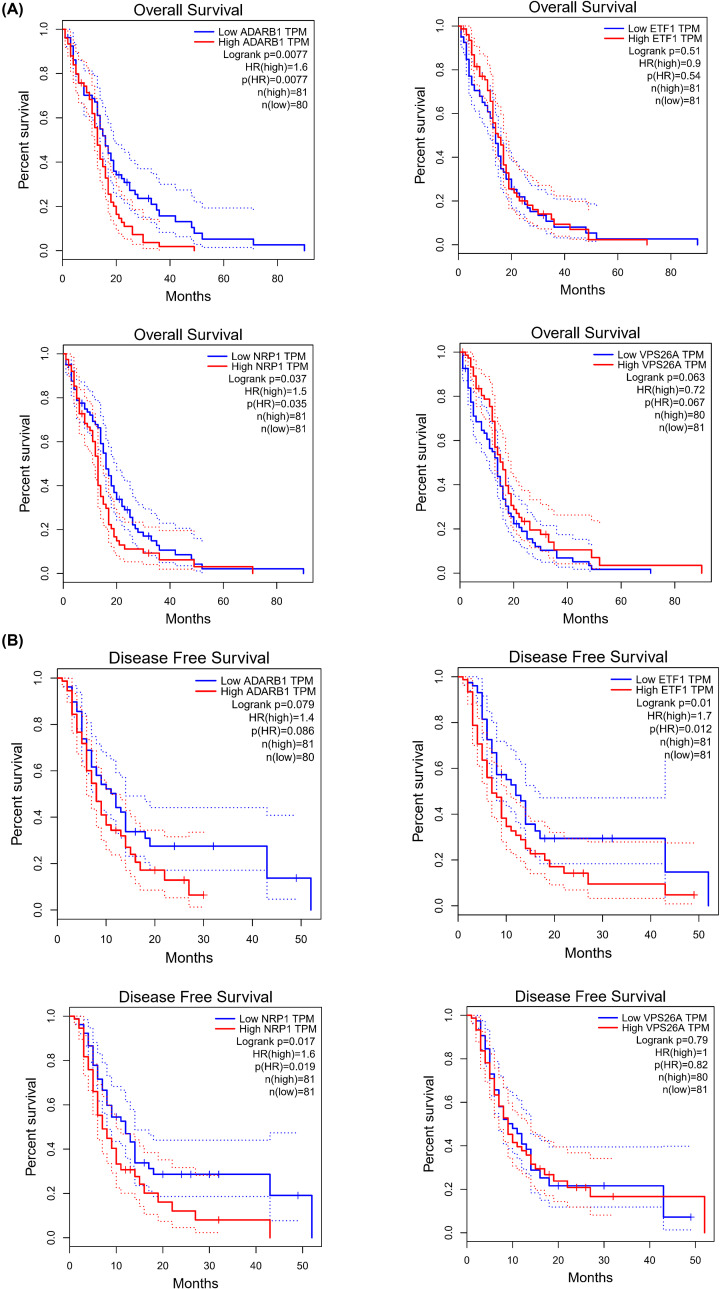
Survival analysis of candidate hub genes (**A**) OS analysis of ADARB1, ETF1, NRP1, VPS26A. (**B**) DFS analysis of ADARB1, ETF1, NRP1, VPS26A.

The overall survival (OS) rate analysis of the GBM, which contained 20-, 40-, 60-month OS and disease-free survival (DFS) analysis, which contained 10-, 20-, were both presented. ADARB1 and NRP1 were negatively correlated with the OS rate in patients with GBM (*P*<0.05) (Figure 5A). ETF1 and NRP1 were negatively correlated with the DFS rate in patients with GBM (*P*<0.05) (Figure 5B).

### Further analysis of key genes

**Figure 6:**
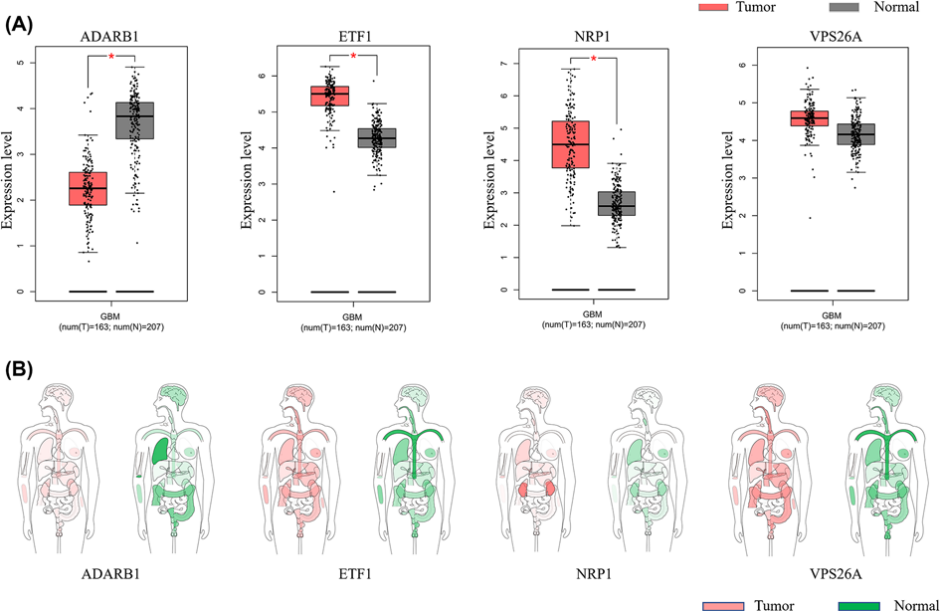
Analysis of candidate hub genes by GEPIA (**A**) Expression of ADARB1, ETF1, NRP1, and VPS26A in GBM tumor tissues and normal brain tissues through GEPIA. (**B**) The median expression of tumor and normal samples in bodymap of ADARB1, ETF1, NRP1, and VPS26A.

We verified the expression of ADARB1, ETF1, NRP1, and VPS26A in GBM tumor tissues and normal brain tissues through GEPIA. Among them, ADARB1 had a lower expression in tumor tissues (*P*<0.05), and ETF1 and NRP1 were highly expressed in tumor tissues (*P*<0.05) (Figure 6A). At the same time, we analyzed the median expression of tumor and normal samples in bodymap (Figure 6B).

**Figure 7:**
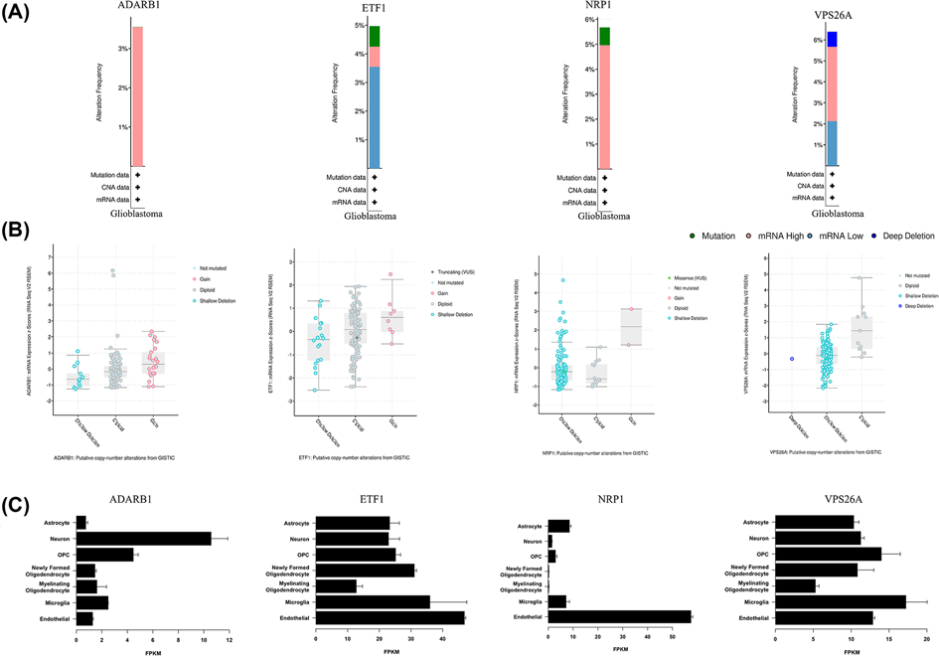
cBioportal analysis and brain RNA-seq analysis of ADARB1, ETF1, NRP1, and VPS26A (**A,B**) cBioportal analysis and putative copy-number alterations from GISTIC. (**C**) Brain RNA-seq analysis showed that the cells of ADARB1 were mainly located in neuron and ETF1, and NRP1 was mainly located in endothelial cells.

We analyzed the Genomic Alteration Types, ADARB1, ETF1, NRP1, and VPS26A, via cBioportal analysis and putative copy-number alterations from GISTIC (Figure 7A,B). Brain RNA-seq analysis showed that the cells of ADARB1 were mainly located in neuron and ETF1, and NRP1 was mainly located in endothelial cells (Figure 7C).

### Verification of hub genes

**Figure 8:**
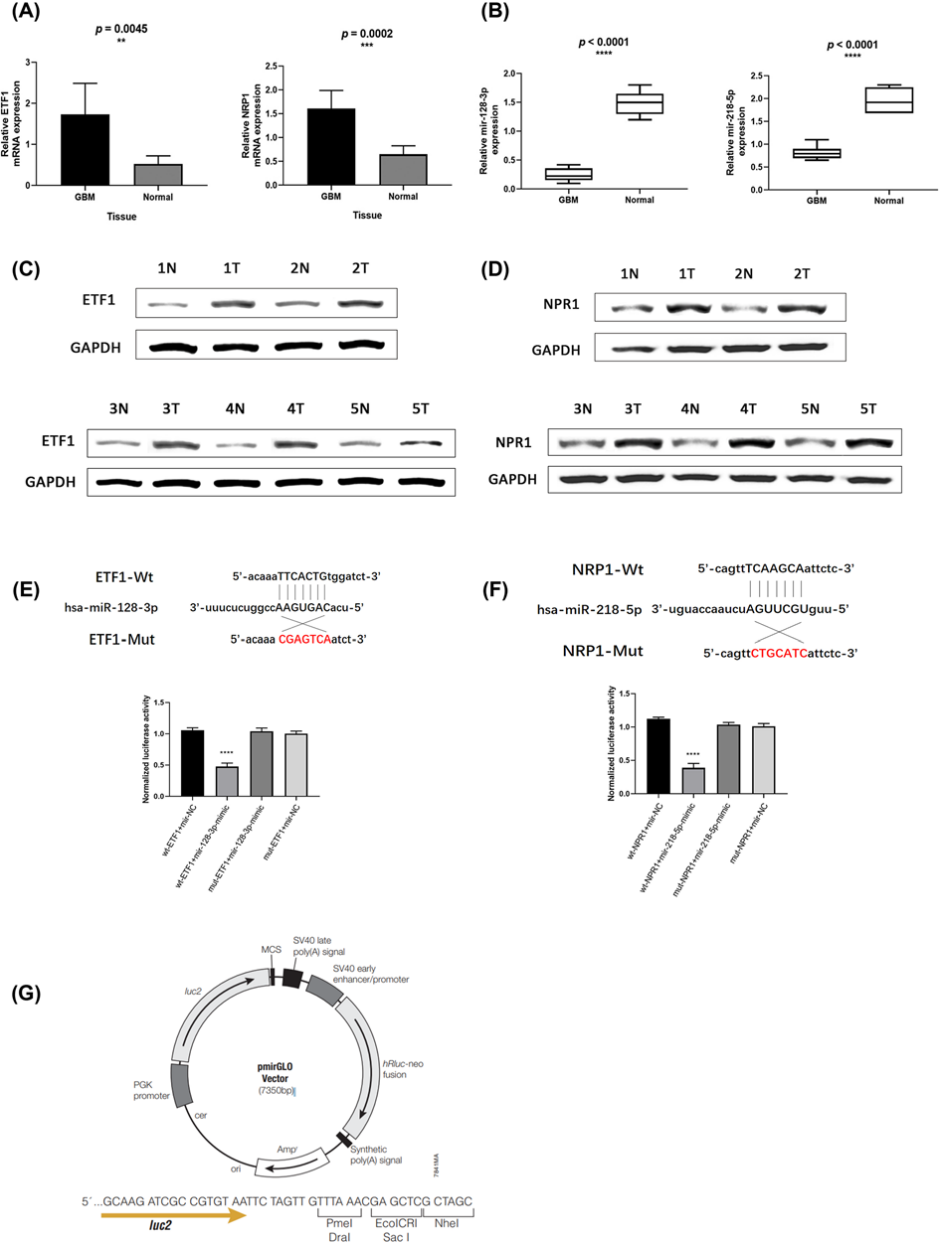
Verification of hub genes and miRNAs (**A,C,D**) ETF1 and NRP1 were highly expressed in GBM tumors (*P*<0.05). (**B**) hsa-mir-128-3p and hsa-mir-218-5p were down-regulated in GBM tissues (*P*<0.05). (**E**–**G**) The luciferase report demonstrated that ETF1 was the target gene of hsa-mir-128-3p and NRP1 was the target gene of hsa-mir-218-5p.

We took both GBM tumor tissues and normal brain tissues for verification analysis. Results of qRT-PCR and Western blot analysis both showed that ETF1 and NRP1 were highly expressed in GBM tumors (*P*<0.05) (Figure 8A,C,D).

RT-PCR showed that both hsa-mir-128-3p and hsa-mir-218-5p were underexpressed in GBM tissues (*P*<0.05) (Figure 8B). The luciferase report demonstrated that ETF1 was the target gene of hsa-mir-128-3p and NRP1 was the target gene of hsa-mir-218-5p (Figure 8E–G).

### Predicted lncRNAs and TFs

**Figure 9:**
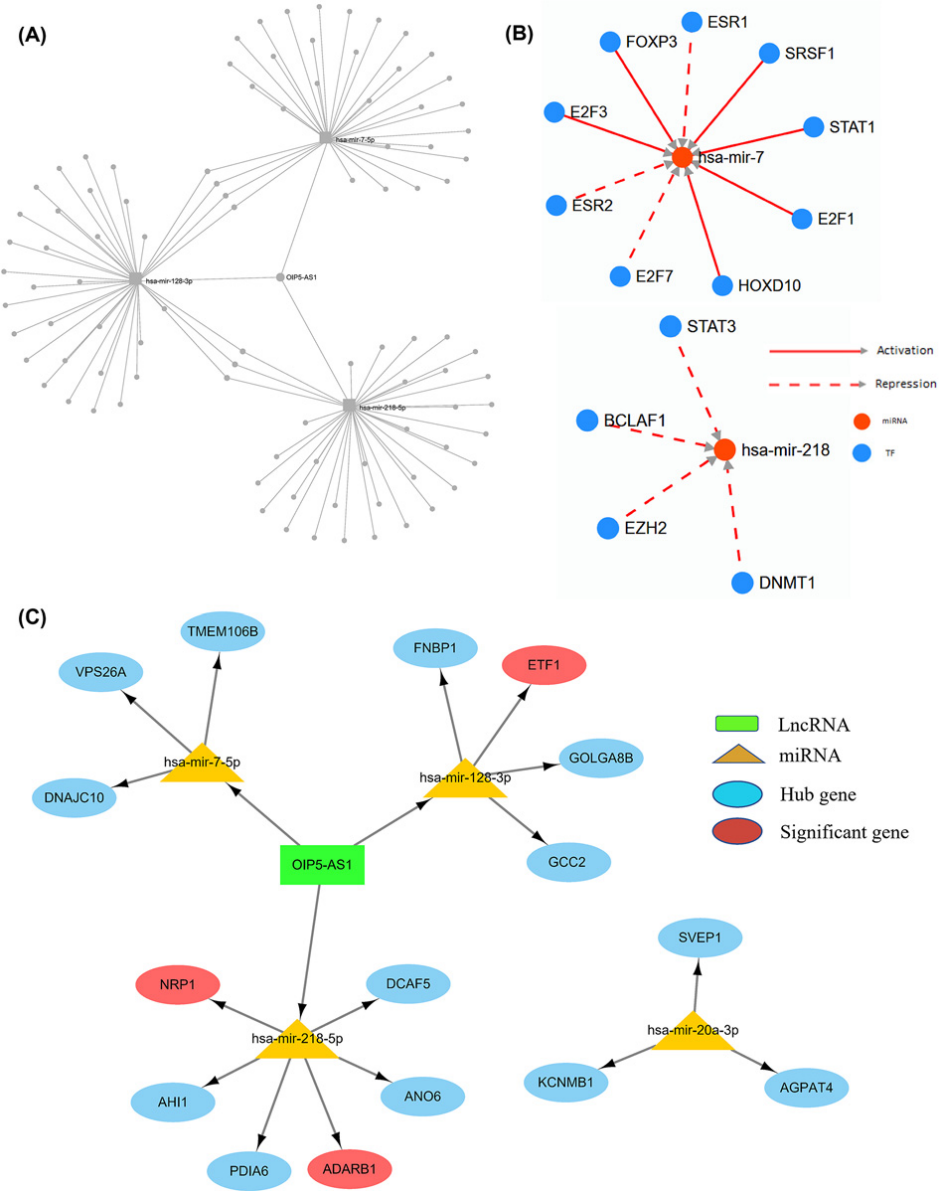
Predicted lncRNAs and TFs (**A**) LncRNAs related to hsa-mir-128-3p, hsa-mir-218-5p, and hsa-mir-7-5p predicted by miRNet. (**B**) TFs related to has-mir-7 and has-mir-218 predicted via TransmiR v2.0. (**C**) A relationship diagram of lncRNA–miRNA–mRNA interaction.

We predicted the lncRNAs related to hsa-mir-128-3p, hsa-mir-218-5p, and hsa-mir-7-5p via miRNet, among which OIP5-AS1 was the predicted lncRNA of three miRNAs (Figure 9A). Some TFs on the upstream of has-mir-7 and has-mir-218 were predicted via TransmiR v2.0 (Figure 9B).

Finally, we used Cytoscape to make a relationship diagram of lncRNA–miRNA–mRNA interaction (Figure 9C).

## Discussion

GBM is a common malignant brain tumor with a poor prognosis [2]. Two subtypes of GBM have been identified in clinical diagnosis. The first subtype is called secondary GBM, which gradually developed from low-grade gliomas. The second subtype is known as primary GBM, which accounts for 90–95% of GBM [24]. GBMs can affect any part of the CNS, especially the deep white matter in the cerebral hemisphere. It is often seen that the frontal and temporal lobes are affected at the same time, with deep and extensive invasion [25]. Early diagnosis, the specificity of key targets, and individualized treatment usually can effectively improve the treatment effect, delay the progress of GBM, and improve the survival time of patients [8]. In this study, hub genes, KCNMB1, AGPAT4, SVEP1, ADARB1, DCAF5, NRP1, PDIA6, AHI1, ANO6, VPS26A, DNAJC10, TMEM106B, ETF1, GCC2, FNBP1, and GOLGA8B, which might be used as targets and biomarkers for treating GBM were selected. In addition, we found miRNAs, hsa-mir-128-3p, hsa-mir-218-5p, hsa-mir-20a-3p, and hsa-mir-7-5p, which might play important roles in the onset and development of GBM. Among them, ETF1, NRP1, hsa-mir-128-3p, and hsa-mir-218-5p were worth paying attention to.

ETF1 is mainly involved in the regulation of translation release factor activity, cytoplasmic translational termination, regulation of translational termination and protein methylation. Abnormal expression of ETF1 can participate in the progression of many diseases. Armakolas et al. found multiple related genes by sequencing and analyzing the blood of patients with breast cancer [26]. Further verification found that ETF1 could be involved in the development of breast cancer and might serve as a biomarker of the HER2 subtype group [26]. Stoddart et al. found that ETF1 could produce potential oncogenic abnormal proteins and may be a potential therapeutic target for myeloid tumors [27]. In addition, Yang et al. investigated the mechanism of treating diabetes mellitus and nephropathy with mesenchymal stem cells with bioinformatics analysis, and further identified ETF1 playing a vital role in the process as a DEG [28]. Wurmser and Emr found that ETF1 was involved in mediating autophagy [29]. There were evidences indicating that inhibiting autophagy might hinder the development of GBM and induce senescence [30]. Furthermore, mechanisms such as apoptosis and autophagy might be involved in affecting the resistance of GBM alkylating agents, and the prognosis of GBM [31]. Similar to the studies mentioned above, we found that ETF1 was highly expressed in patients with GBM. Survival analysis found that patients with high expression of ETF1 have poor prognoses. At the same time, we had verified that ETF1 was highly expressed in patients with GBM through UCSC, GEPIA, Western blot, and RT-PCR. Also, we found that ETF1 was the target gene of hsa-mir-128-3p by luciferase detection. We speculated that ETF1-hsa-miR-128-3p participated in the generation and development of GBM by processes such as regulating nuclear transcriptional mRNA catabolism, cytoplasmic translation, and autophagy. ETF1 and its related molecules may serve as targets for early diagnosis and specific treatment of GBM. The related molecular mechanism is worth further exploration.

NRP1 mainly engages in vascular endothelial growth factor (VEGF)-activated receptor activity, protein kinase binding, angiogenesis, neuron migration, positive regulation of endothelial cell proliferation, positive regulation of phosphorylation, and negative regulation of neuron apoptotic process [32]. The abnormal expression of NRP1 is involved in various pathophysiological processes of the body. Li et al. found that miR-1247 regulated the apoptosis and inactivation of the Wnt/β-catenin pathway in osteosarcoma, and Nrp1 could inhibit this pathway and then go against the prognosis of patients with osteosarcoma [33]. Pang et al. noticed that as a target gene for miR-628, Nrp1 could inhibit apoptosis by promoting proliferation, migration, and invasion, so as to participate in the development of gastric cancer, suggesting that it may be a therapeutic target for gastric cancer [34]. Frankel et al. believed that NRP1 and chondroitin sulfate modified Nrp1 (Nrp1-CS) may participate in the invasion of GBM cells by affecting tyrosine phosphorylation, indicating that it may be a potential diagnostic and therapeutic target [35]. In addition, Nasarre et al. found that Nrp1 antagonist peptides that target the Nrp1 transmembrane domain can effectively prevent the growth of rat and human gliomas in the body by inhibiting VEGF signaling and may prolong the survival time of patients with glioma [36]. Hamerlik et al. considered that directly inhibiting VEGFR2 kinases can block the highly dynamic VEGF-VEGFR2-Nrp1 pathway, thereby effectively inhibiting the development of GBM and improving the prognosis [37]. Furthermore, Sun et al. found that Nrp1 is a receptor for glial cell line-derived neurotrophic factor (GDNF) in glioma cells and could be a potential therapeutic target for GBM [38]. In fact, VEGF-related drugs could effectively suppress the vessel growth, which in turn slowed the progression and metastasis of GBM. However, short-term effects are usually better, and long-term effects are still limited. Tumor recurrence is commonly seen in GBM [39]. In anti-angiogenic therapy, the mechanisms driving acquired resistance and tumor recurrence remain unclear. And the molecular mechanism of VEGF–Nrp1 interaction is insufficiently reported [40]. Ma et al. found that overexpression of Nrp1 promoted the expression and secretion of high mobility group box 1 (HMGB1) and endothelial inflammation, and that the mitogen-activated protein kinase (MAPKs) pathway was involved in this process [41]. Consistent with the above studies, we also found that NRP1 was highly expressed in GBM patients. The survival analysis found that patients with higher NRP1 expression have a worse prognosis. Then, we verified the high expression of NRP1 in GBM patients by UCSC and GEPIA, as well as the high expression of NRP1 in GBM patients by Western blot and RT-PCR. Also, we found that NRP1 was the target gene of hsa-mir-218-5p by luciferase detection. We speculated that NRP1 and hsa-mir-218-5p participated in the generation and development of GBM by processes such as regulating angiogenesis, apoptosis, and phosphorylation. NRP1 and its related molecules could serve as targets for early diagnosis and treatment of GBM. The specific molecular mechanism and upstream and downstream regulatory networks involved in the occurrence and development of GBM need to be further explored.

Although a rigorous bioinformatics analysis was performed in the present study, there were still some shortcomings. First, the sample size in the dataset was small. The sample size needed to be further expanded to obtain more accurate results. Second, this article only conducted a small sample verification. It would necessary to use a larger number of clinical samples and animal experiments for comprehensive verification in order to better understand the molecular mechanism of GBM. Third, we predicted lncRNAs and TFs related to miRNAs. Howerver, the mechanism of these molecules involved in the generation and development of GBM still needs further experimental verification.

## Conclusions

Bioinformatics technology could be a useful tool to find biomarkers of GBM. DEGs and DEMs were found between GBM tumor tissues and normal cerebral tissues, which could engage in the related mechanism of the generation and development of GBM. These biomarkers may be used as targets for early diagnosis and specific treatment.

## Data Availability

The datasets used and/or analyzed during the current study are available from the corresponding author on reasonable request.

## Abbreviations

BP: biological process
CNS: central nervous system
DAVID: Database for Annotation, Visualization and Integrated Discovery
DEG: differentially expressed gene
DEM: differentially expressed miRNA
DFS: disease-free survival
ETF1: eukaryotic translation termination factor 1
FC: fold change
GBM: glioblastoma
GO: Gene Ontology
GPEC: Gene Prioritization by Evidence Counting
KEGG: Kyoto Encyclopedia of Genes and Genomes
MF: molecular function
NRP1: neuropilin 1
OS: overall survival
PPI: protein–protein interaction
STRING: Search Tool for the Retrieval of Interacting Genes
TCGA: The Cancer Genome Atlas
TF: transcription factor
VEGF: vascular endothelial growth factor
